# Metformin in polycystic ovary syndrome: unraveling multi-stage therapeutic mechanisms from puberty to long-term health outcomes

**DOI:** 10.3389/fphar.2025.1654372

**Published:** 2025-08-18

**Authors:** Weiwei Zeng, Yuning Luo, Juanfeng Ou, Dali Gan, Min Huang, Brian Tomlinson, Yiming Jiang

**Affiliations:** ^1^ Department of Pharmacy, Shenzhen Longgang Second People’s Hospital, Shenzhen, Guangdong, China; ^2^ Institute of Clinical Pharmacology, School of Pharmaceutical Sciences, Sun Yat-sen University, Guangzhou, Guangdong, China; ^3^ Faculty of Medicine, Macau University of Science and Technology, Macau, China; ^4^ School of Pharmaceutical Sciences, Sun Yat-sen University, Guangzhou, Guangdong, China

**Keywords:** metformin, polycystic ovary syndrome, infertility, mechanism, drug therapy

## Abstract

Polycystic ovary syndrome (PCOS) represents a prevalent endocrine disorder affecting reproductive-aged women worldwide, characterized by a variety of reproductive, metabolic, and psychological manifestations. This condition disrupts menstrual cycles and fertility, and significantly compromises quality of life, while increasing the risk of severe health consequences, including cardiovascular diseases and endometrial carcinoma. Although the precise etiology of PCOS remains elusive, genetic and environmental factors are thought to contribute to its pathogenesis. In recent years, the escalating global prevalence of PCOS has been observed, and pharmacological intervention has become the primary treatment approach. Metformin, an insulin sensitizer, has emerged as a valuable treatment option in PCOS management. Multiple studies have suggested that metformin have a positive impact on puberty problems, pregnancy complications, and long-term health outcomes in women with PCOS. However, persistent controversies surround its therapeutic efficacy and underlying molecular mechanisms. This review systematically examines the mechanisms of metformin in ameliorating PCOS-associated infertility, with particular emphasis on its pleiotropic effects across critical life stages—from pubertal development through pregnancy to long-term health outcomes, thereby providing valuable insights into the clinical application of metformin in the treatment of PCOS.

## 1 Introduction

Infertility stands as a significant global health issue, impacting numerous families and nations worldwide. According to the World Health Organization (WHO), approximately 17.5% of adults of reproductive age experience infertility. Female reproductive system-related infertility may stem from diverse etiological factors, including abnormalities of the uterus, ovaries, fallopian tubes, and endocrine system. Among ovarian pathologies contributing to female infertility, polycystic ovary syndrome (PCOS) emerges as a predominant causative condition. Originally termed Stein-Leventhal syndrome, PCOS represents a multisystem disorder characterized by intricate associations with metabolic dysregulation and hypothalamic-pituitary-ovarian axis (HPOA) dysfunction, posing substantial threats to fertility during women’s prime reproductive years (Gleicher et al., 2022). According to the 2023 epidemiological survey, the prevalence of PCOS among women of reproductive age is 8%–13%, indicating that PCOS is more prevalent among women in their peak reproductive years, typically between 20 and 30 years of age (Forslund et al., 2022).

PCOS typically manifests during adolescence and is diagnostically characterized by three main features: namely long-term ovulatory dysfunction, hyperandrogenism (HA), and polycystic ovarian morphology, which form the basis of the diagnostic criteria for PCOS (Siddiqui et al., 2022). Multiple studies suggested that PCOS may further induce serious complications such as diabetes, hypertension, endometrial cancer (EC) and preeclampsia without timely intervention, which can severely affect the quality of life and long-term health outcomes of patients (Cooney and Dokras, 2018). Early identification and effective management of PCOS are crucial to improving the fertility and overall health status of patients (Gilbert et al., 2018; Krentowska et al., 2021).

Current clinical practice recognizes that there is no one-size-fits-all therapeutic approach for PCOS management. However, weight loss through dietary control and regular exercise is considered a fundamental and critical treatment measure, especially in obese or overweight individuals with PCOS. Approximately 30% of obese patients with PCOS may resume ovulation after weight loss and increase the likelihood of successful pregnancy (Teede et al., 2018; Arasu et al., 2019). Pharmacological intervention is the most common therapeutic strategy. For patients with PCOS without immediate fertility goals, oral contraceptives or progestins (synthetic progesterone analogs) are routinely prescribed. Those seeking pregnancy may utilize ovulation-inducing agents such as clomiphene citrate, metformin, or gonadotropins to stimulate follicular maturation and improve conception likelihood. Patients with PCOS presenting with hyperandrogenic manifestations such as hirsutism, acne and alopecia may benefit from anti-androgen therapies (e.g., spironolactone, flutamide, or finasteride) that inhibit androgen synthesis or receptor activity, thereby ameliorating cutaneous and pilosebaceous symptoms (Lizneva et al., 2016). In addition, for PCOS patients with insulin resistance (IR), metformin can effectively reduce hyperglycemia and hyperinsulinemia, improve ovulatory function, reduce androgen levels, control weight and waist circumference gain, and mitigate the risks of diabetes mellitus and cardiovascular disease (Notaro and Neto, 2022). The detailed pharmacological therapeutic strategies are shown in Table 1.

**TABLE 1:** Therapeutic drug strategies for patients with polycystic ovary syndrome.

Treatment objective	Medication	Pharmacological effect	Precautionary note
Promoting Fertility	Metformin	Improve IR metabolic abnormalities, and prevention cardiovascular diseases	Regular monitoring of liver and kidney function
	Clomiphene/Gonadotropin	Prevention of perinatal complications and fetogenic diseases	Infertility should be ruled out before using the drug
Adjustment of menstrual cycle	Contraceptives/Short-acting COC	Suppresses LH, ovulation and Protecting the endometrium to prevent EC	Do not increase or decrease the dose, if miss, take it immediately (within 24 h)
	Spironolactone & Finasteride	Reduces androgen levels, improves hirsuteness and acne	Contraception is suggested for the duration of the medication

Abbreviations: IR: insulin resistance; COC: combined oral contraceptives.

Current mechanistic studies indicated that IR is a key factor in the pathogenesis and progression of PCOS. During adolescence, high insulin levels caused by IR may disrupt the gonadotropin-releasing hormone (GnRH) signaling pathway and induce HA through multiple pathways, thereby affecting the establishment of ovulatory function. IR exerts important regulatory effects on ovarian function, endometrial health and glucose-lipid metabolism in patients with PCOS, serving as a primary contributor to infertility and unfavorable long-term outcomes in reproductive-aged females. Improving IR is crucial for the treatment of patients with PCOS. Metformin, an established insulin-sensitizing agent, has gained widespread clinical application in PCOS treatment. Recent clinical and preclinical studies have demonstrated that metformin not only ameliorates insulin sensitivity, but also facilitates glucose-lipid reverse transport, alleviates hormone imbalances induced by glucose metabolic irregularities, and ameliorates symptoms associated with hormonal dysregulation in patients with PCOS (Shen et al., 2022; Yang et al., 2022). Significantly, metformin has been demonstrated to normalize pulsatile GnRH secretion by reducing circulating insulin levels. This indirect mechanism facilitates the induction of ovulation, thereby addressing infertility caused by ovulatory dysfunction. Moreover, metformin has been demonstrated to independently regulate menstrual cycles and ovulation, which further enhances fertility (Shao et al., 2023). Additionally, studies have found that metformin can improve cardiovascular function and the outcomes for offspring during pregnancy (Panagiotopoulou et al., 2020). Despite its demonstrated efficacy and therapeutic promise, there is still uncertainty regarding its role in the metabolic regulation of PCOS, especially concerning the long-term effects on reproductive metabolic disorders. Hence, this review attempts to provide insight into the mechanisms by which metformin ameliorates adverse reproductive outcomes in PCOS. In this review, we also aim to elucidate the effects of metformin on adolescence, pregnancy, and the long-term health of patients with PCOS, so as to meet the diverse clinical treatment requirements of individuals with PCOS and to improve the fertility of females in their reproductive years.

## 2 Mechanisms of metformin alleviating anovulatory infertility in polycystic ovary syndrome

**FIGURE 1:**
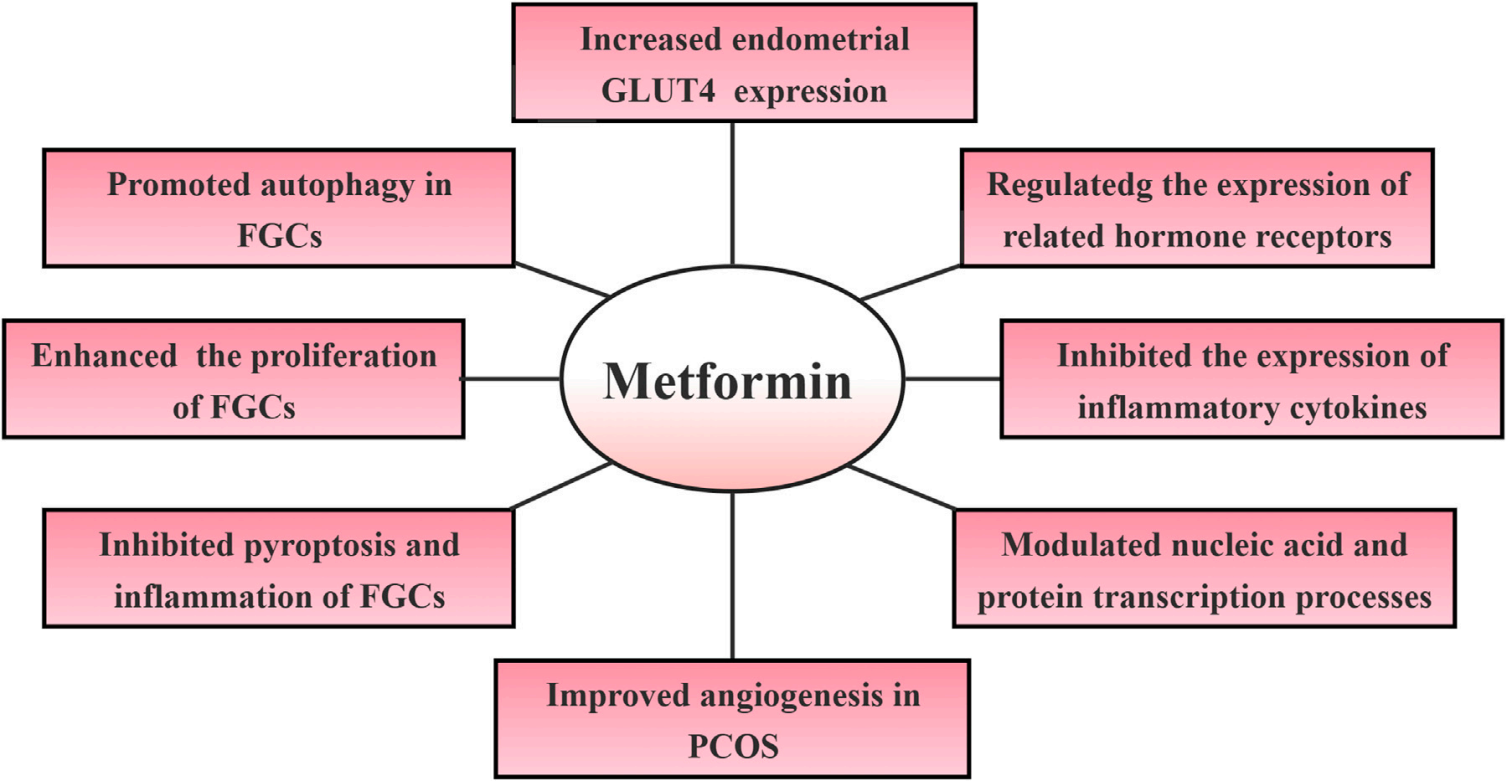
Mechanisms of metformin regulation of infertility in polycystic ovary syndrome. Abbreviations: GLUT4: glucose transporter protein 4; FGCs: follicular granulosa cells; PCOS: polycystic ovary syndrome.

For females of childbearing age, the most significant consequence of PCOS is infertility, for which the pathogenesis is still unclear. Current studies have hypothesized that infertility in patients with PCOS may be due to the following reasons (Kicińska et al., 2023). The primary pathophysiological mechanism involves dysregulated luteinizing hormone (LH) secretion coupled with diminished follicle-stimulating hormone (FSH) levels, culminating in abnormally elevated androgen concentrations and IR. This endocrine imbalance precipitates premature follicular development and stagnation or even cessation of ovulation (Rosenfield and Ehrmann, 2016). Secondly, the persistently high level of LH secretion promotes ovarian stromal vascular proliferation, increased stromal blood flow, enhanced oxygen metabolism, and increased production of reactive oxygen species (ROS), triggering oxidative stress, which adversely affects oocyte maturation, fertilization, embryo development, and the progress of pregnancy (Awonuga et al., 2023). Consequently, due to impaired follicular maturation and the absence of early ovulation, individuals with PCOS cannot cyclically shed the subendometrial layer (Li et al., 2022). The estrogen stimulation induces progressive thickening of the endometrial lining, leading to atypical growth or even cancer. Therefore, high-quality embryos and optimal endometrial receptivity are the prerequisites for a successful pregnancy. Optimizing pregnancy outcomes in patients with PCOS necessitates a multifaceted therapeutic approach centered on promoting follicular maturation, inducing ovulation, and enhancing endometrial receptivity. Research has demonstrated that metformin exhibits favorable regulatory effects in promoting follicular development, inducing ovulation, and enhancing endometrial receptivity. Its specific regulatory mechanism is shown in Figure 1.

### 2.1 Metformin promotes follicular development and ovulation induction

#### 2.1.1 Promoted autophagy in follicular granulosa cells

Promoting follicular development and inducing ovulation are cornerstones in the management of infertility in PCOS. Follicular granulosa cells (FGCs) play an important role in follicle formation (Owens et al., 2019). FGCs synthesize and secrete hormones and cytokines that contribute to normal follicle development and maturation. Folliculogenesis is predominantly regulated by the proliferation and apoptosis of FGCs. In PCOS, the reduced quantity and impaired function of FGCs can lead to significant disruptions in follicular development, thereby increasing the risk of infertility. The mammalian target of rapamycin (mTOR), a key protein downstream of the phosphatidylinositol 3-kinase/protein kinase (PI3K/AKT) pathway, is closely associated with insulin metabolic dysfunction and exerts a substantial impact on the growth and proliferation of FGCs. Additionally, mTOR could inhibit apoptosis and promote angiogenesis, thus playing a crucial role in folliculogenesis (Kim and Guan, 2015; Ersahin et al., 2015). Aziz et al. found defective insulin levels and impaired PI3K/AKT/mTOR signaling pathways in the ovary and endometrium of patients with PCOS (Aziz et al., 2020). Xu et al. observed that metformin improved PCOS in rat models by downregulating autophagy in FGCs. At the same time, metformin reduced H_2_O_2_-induced oxidative stress and autophagy levels in FGCs, via the PI3K/AKT/mTOR signaling pathway (Xu et al., 2022). This provides evidence for reducing excessive autophagy in ovarian granulosa cells and improving PCOS symptoms. Similarly, other researchers have found that dimethoate, an acetylcholinesterase inhibitor, induces ovulation by promoting autophagy in FGCs (Bhansali et al., 2020).

#### 2.1.2 Promoted the proliferation and differentiation of follicular granulosa cells

Growth differentiation factor 9 (GDF-9) and bone morphogenetic protein 15 (BMP-15), members of the transforming growth factor-β (TGF-β) superfamily, are crucial for the proliferation and differentiation of granulosa cells and oocytes, impacting oocyte development and quality, and modulating ovarian function (Sanfins et al., 2018). Dysregulation of their expression can result in aberrant follicular development and changes in the structure of the zona pellucida in individuals with PCOS, playing a significant role in infertility. BMP-15 may be exert pathophysiological relevance to IR in patients with PCOS through canonical activation of the Smad1/5/8 signaling pathway (Chang et al., 2016). Iwata et al. demonstrated that metformin administration could enhance the progesterone production induced by forskolin and FSH in both human FGCs and rat primary FGCs. In human FGCs, metformin treatment was demonstrated to increase the expression of inhibitory Smad6 while inhibiting the phosphorylation of BMP-15-activated Smad1/5/9 (Iwata et al., 2018). Metformin can directly influence progesterone production and promote follicular growth by modulating the BMP system in FGCs. Furthermore, studies have suggested that the therapeutic synergy between sitagliptin and metformin co-administration can increase mRNA and protein expression levels (Daneshjou et al., 2022). Similarly, Bee pollen (BP) and metformin can synergistically improve the proliferation and inhibit apoptosis of follicular FGCs (Naseri et al., 2022). Zhang et al. also found that the combination of metformin and Diane-35 improved the oestrous cycle disorders, increased the proliferation and differentiation of ovarian FGCs, and inhibited cell apoptosis in PCOS rats. The mechanism may be related to metformin regulating glycolysis-related mediators to improve ovarian energy metabolism (Zhang et al., 2020).

#### 2.1.3 Inhibited pyroptosis and inflammation of follicular granulosa cells

Furthermore, researchers have discovered that metformin has the potential to enhance follicle development and induce ovulation by suppressing pyroptosis and inflammation of follicular granulosa cells. This effect may be attributed to its modulation of molecular pathways involving matrix metalloproteinase (MMP)-2 and MMP-9 expression, as well as nicotinamide adenine dinucleotide phosphate (NADPH) oxidase 2 gene *(NOX2)* levels. In their study, Chen et al. observed the impact of metformin on the H19 and AMPK signaling pathways in serum samples from rats with PCOS. They observed that metformin could downregulate the expression of MMP-9/MMP-2 and mTOR while upregulating the expression of protein kinase B and adenosine monophosphate-activated protein kinase (AMPK), thereby inhibiting ovarian granulosa cells dysfunction and promoting their recovery of follicle function (Chen et al., 2019). In addition, the researchers investigated metformin’s regulatory effects on pyroptotic pathways in FGCs, elucidating its molecular mechanism of action. Experimental evidence from *in vitro* studies utilizing human granulosa tumor-derived cell models demonstrates that metformin intervention effectively downregulates multiple components of the LPS-activated inflammatory cascade, including LPS-induced *miR-670-3p*, *NOX2*, *NLRP3*, *ASC*, and *GSDMD-N*, as well as reducing cellular caspase-1 activity, ROS production, and secretion of inflammatory factors. Additionally, upregulation of *NOX2* has been shown to enhance the anti-pyroptotic and anti-inflammatory effects of metformin. The study also revealed that *miR-670-3p* plays a direct role in the *NOX2* gene and inhibits its expression, while overexpression of *NOX2* significantly enhances the anti-pyroptosis and anti-inflammatory effects of metformin. Overall, metformin may inhibit pyroptosis of human granulosa-like tumor cell line cells through the *miR-670-3p/NOX2/ROS* pathway (Zhou et al., 2023).

#### 2.1.4 Improved angiogenesis in polycystic ovary syndrome

Among other heterogeneous features, abnormal angiogenesis is one of the characteristics of PCOS. Vascular endothelial growth factor (VEGF) is a major factor in embryonic development and regulation of physiological angiogenesis for reproductive function. Metformin has been identified to significantly improve angiogenesis in PCOS. Di et al. investigated the effect of metformin on ovarian angiogenesis by constructing a dehydroepiandrosterone-induced PCOS rat model. The results revealed that metformin treatment increased the percentage of follicular accumulation and luteinization, with a significant reduction in ovarian cysts in PCOS rats. Moreover, the levels of vascular VEGF, angiopoietin, and platelet-derived growth factor (PDGF)-B&D were restored. The normalization of these markers facilitated follicular development and ovulation in PCOS rats (Di Pietro et al., 2015). Other researchers have discovered that metformin promoted an increase in follicle number and improved the expression of VEGF-A and gonnective tissue growth factor (CTGF-1), and serum levels of estrogen, testosterone, and insulin were significantly adjusted (Attia et al., 2019). Mahamed et al. explored the effect of metformin on 17-hydroxylase expression in the ovarian tissue of PCOS rats, as well as its effects on follicular dynamics and proliferative parameters in PCOS rats. They found that metformin can not only significantly improve 17-hydroxylase expression in ovarian structures, reduce body weight, increase glucose and insulin levels in PCOS rats, but also promote the proliferation of sphincteric interstitial and mesenchymal cells and improve angiogenesis, which consequently promotes follicular development and ovulation (Mahamed et al., 2018).

### 2.2 The regulatory mechanism of metformin on endometrial receptivity

**FIGURE 2:**
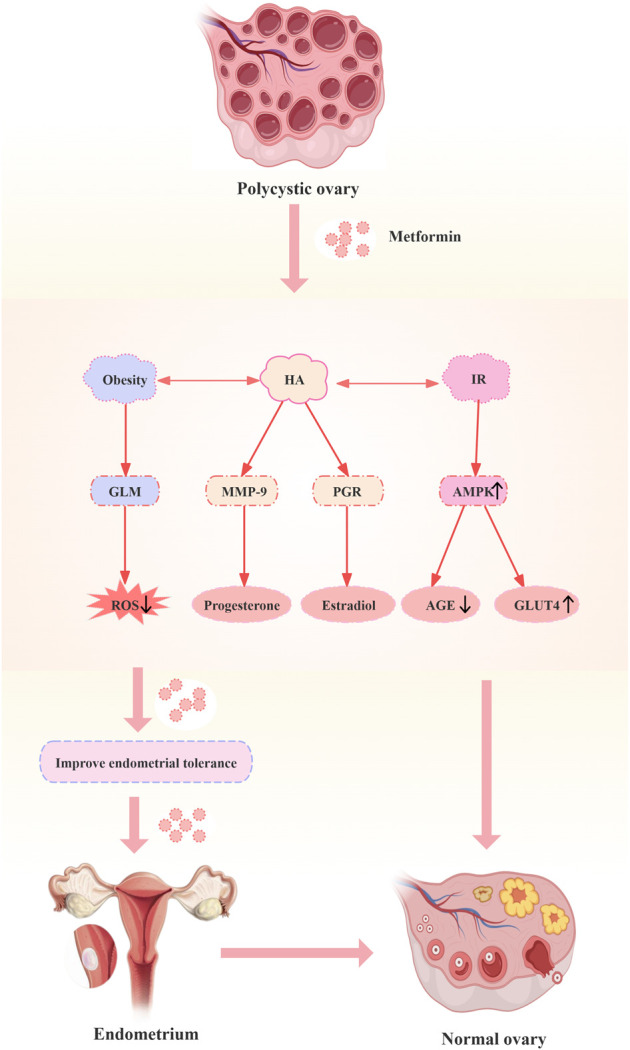
Mechanisms of metformin modulation of endometrial tolerance in polycystic ovary syndrome. Abbreviations: HA: hyperandrogenemia; IR: insulin resistance; GLM: glucose-lipid metabolism; MMP-9: matrix metalloproteinase; PGR: progesterone; AMPK: adenosine monophosphate-activated protein kinase; ROS: reactive oxygen species; AGE: advanced glycation end product; GLUT4: glucose transporter protein 4.

A good endometrium allows the blastocyst to attach, penetrate, and induce local decidualization of interstitial cells, thus providing an ideal environment for implantation of the fertilized egg and offering support for the growth of the embryo. Therefore, decreased endometrial receptivity is also one of the vital factors leading to infertility or miscarriage in patients with PCOS. Abnormal autophagy, HA, IR, obesity, impaired ecdysis and aberrant expression of relevant tolerance markers were found to have an impact on endometrial receptivity in patients with PCOS. Researchers found that metformin could regulate endometrial tolerance by increasing endometrial glucose transporter protein 4 (GLUT4) expression, modulating the expression of related hormone receptors to affect endometrial transformation, modifying autophagy, as well as modulating DNA/histone modifications and micro RNAs (miRNAs) for transcriptional regulation. The mechanisms by which metformin modulates endometrial tolerance in PCOS are shown in Figure 2.

#### 2.2.1 Increased endometrial glucose transporter protein 4 expression

AMPK is a major energy receptor. It mainly regulates glucose and lipid metabolism to maintain intracellular energy balance. In patients with PCOS, endometrial GLUT4 expression is decreased, and glucose transport and utilization are impaired, leading to endometrial energy metabolism disorders, which in turn affect the establishment of endometrial tolerance. It has been found that metformin can improve glucose transport and utilization by activating AMPK and promoting GLUT4 expression. This provides sufficient energy for endometrial thickening to promote endometrial metamorphosis and furnishes a potent oxygen environment for embryo implantation. Meanwhile, metformin effectively inhibited endometrial thickening in PCOS rats, while attenuating the overexpression of GLUT4 in the endometrium (Zhai et al., 2012). Furthermore, Ferreira et al. discovered that the combination of metformin and insulin led to a significant increase in the expression of growth factor-associated proteins, impacting the proliferation of endometrial stromal cells (Ferreira et al., 2022). Additionally, Liu et al. demonstrated that metformin could potentially hinder endometrial proliferation through the regulation of *lncRNA-MEG3/miR-223/GLUT4* and *lncRNA-SNHG20/miR-4486/GLUT4* signaling pathways. However, it was observed that despite the improvement in blood glucose levels in insulin-resistant pregnant rats, metformin also aberrantly activated AMPK and stimulated glycogenolysis in the endometrium (Liu J. et al., 2022). Different researchers have reported varying findings, and more experimental data are needed to verify the effects.

#### 2.2.2 Modulation of the expression of relevant hormone receptors affects endometrial metamorphosis

The collaborative function of progesterone (P4) and estradiol (E2) is essential for establishing endometrial tolerance during the implantation window period in the natural reproductive cycle of mice (Gudelska et al., 2023). Yang et al. identified impaired leukemia inhibitory factor/signal transduction (LIF/STAT3) pathway and disrupted endoplasmic reticulum stress in mice treated with excessive P4, leading to potential inhibition of embryo implantation and uterine transformation (Liang et al., 2018). Patients with PCOS demonstrate dysregulated expression of steroid hormone receptor and homeobox A10 (HOXA10), which play crucial roles in the process of embryo implantation within the endometrium. Ohara et al. conducted a study to investigate the impact of metformin on the levels of androgen receptor and HOXA10 expression in individuals with PCOS. The results revealed a decrease in androgen receptor expression in both epithelial and stromal cells following 3 months of metformin treatment, while HOXA10 expression increased in stromal cells. Furthermore, metformin was shown to mitigate the effects of testosterone-induced hormone receptor expression, and restore testosterone and HOXA10 expression levels (Ohara et al., 2021).

#### 2.2.3 Others

Metformin influences the production of advanced glycation end products (AGEs) and the generation of ROS through the activation of AMPK, inhibition of the receptor for advanced glycation end products/nuclear factor kappa B (RAGE/NF-κB) signaling pathway, and modulation of gene expression. The accumulation of AGEs is known to be linked with IR and the aging process, which has the potential to hinder folliculogenesis and diminish endometrial receptivity. Furthermore, metformin has been shown to improve endometrial tolerance through its actions on the mitochondrial respiratory chain, activation of AMPK, suppression of NF-κB, AGE production, and downregulation of inflammatory pathways (Jinno et al., 2021). It was also found that metformin constrains lipopolysaccharide (LPS)-induced chemokine expression via AMPK and NF-κB signaling pathways (Ye et al., 2018).

In addition, metformin could regulate DNA/histone modifications and miRNAs to carry out transcriptional regulatory processes (Alimoradi et al., 2021). Zhai et al*.* analyzed the expression of HOXA10 and integrin beta-3 (ITGB3) in the endometrium of individuals treated with metformin compared to a control group, as well as the regulatory influence of miRNAs, miR-1910-3p and miR-491-3p, on HOXA10 and ITGB3. The findings indicated that metformin treatment led to enhanced endometrial tolerance through the downregulation of tolerance-related miRNAs, specifically miR-1910-3p and miR-491-3p, resulting in elevated expression of endometrial tolerance markers HOXA10 and ITGB3 in patients with PCOS. Notably, the effects of metformin exhibited a significant dose-dependent relationship (Zhai et al., 2019).

Autophagy is a tightly regulated catabolic process essential for maintaining intracellular stability. In the realm of reproduction, appropriate levels of autophagy are crucial for oocyte development and maturation, as well as for preventing endometrial metaplasia (Devis-Jauregui et al., 2021). Research has demonstrated that decreasing autophagy via the AMPK/mTOR pathway could result in abnormal endometrial metaplasia in early pregnancy in mice, potentially affecting pregnancy outcomes. Metformin, as an AMPK activator, promotes autophagy by enhancing the AMPK/mTOR signaling pathway (Abdellatif et al., 2018). Furthermore, metformin has the potential to modulate glucose-lipid metabolism (GLM) to mitigate oxidative stress, suppress inflammatory reactions, shield cells from oxidative damage, and enhance the fertility of the endometrium. It has been postulated that metformin may also boost endometrial receptivity by alleviating immune responses and complement activation triggered by oxidative stress (Chung et al., 2017). Nevertheless, more experimental data are required to verify these effects.

## 3 Impact of metformin on different stages of polycystic ovary syndrome

### 3.1 Impact of metformin on adolescent patients with polycystic ovary syndrome

The prevalence of adolescent PCOS has shown a notable increase in recent years. The definitive diagnosis of adolescent PCOS poses a significant challenge due to the substantial overlap of symptoms with normal physiological characteristics of adolescence. This complicates the differentiation between pathological signs and typical developmental features. The absence of standardized diagnostic criteria contributes to variations in reported prevalence rates. Common symptoms among adolescent patients include irregular menstruation, oligomenorrhea, and amenorrhea, with the latter being the most frequently observed symptom, followed by oligomenorrhea and abnormal uterine bleeding (AUB) (Meczekalski et al., 2023; Kostopoulou et al., 2020). The prevalence of PCOS detected by ultrasound can be as high as 40% in adolescents with significant symptoms of PCOS. Additionally, the occurrence of hirsutism, acne, and ovarian polycystic changes was significantly higher in adolescent patients with AUB compared to those with regular menstrual cycles. Research findings suggest a potential link between the physical manifestations of PCOS and adverse psychological effects, including decreased self-esteem and increased anxiety in pubescent females, which may exacerbate mood disorders and reduce the overall quality of life in adolescent patients with PCOS. As a result, adolescents with PCOS tend to have a higher prevalence of anxiety and depression (Jiskoot et al., 2022; Nasiri-Amiri et al., 2023). In summary, improving obesity, hirsutism, acne, and other related physical symptoms plays a crucial role in the management of adolescent patients with PCOS. (Che et al., 2023).

Treatment at various stages can help reduce the short-term and long-term impacts of PCOS. Lifestyle modifications involving exercise and dietary changes are considered the initial treatments for patients with PCOS and should be incorporated into the patient’s overall management plan (Mizgier et al., 2021; Reiser et al., 2022). According to national clinical guidelines, metformin is strongly recommended as a first-line treatment for adolescent PCOS, either as a standalone therapy or in combination with oral contraceptives. This treatment is aimed at reducing testosterone levels, improving symptoms such as hirsutism and acne, and restoring regular menstrual cycles (Teede et al., 2023). Fraison et al. also found that metformin combined with oral contraceptives effectively improves hirsutism, acne, and menstrual irregularities in adolescent PCOS (Fraison et al., 2020). Similarly, other researchers have reported comparable treatment effects (Yen et al., 2021). Researchers were interested in investigating the potential variations in the effectiveness of metformin treatment among different stages of adolescence and phenotypes. Ibanez et al. focused on changes in weight, hirsutism scores, menstrual cycles, endocrine-metabolic profiles, and other factors in adolescent patients with PCOS with low birth weight (LBW) and precocious pubarche (PP) who received metformin treatment at different stages. The results showed that early administration of metformin was associated with a significant increase in height in 15-year-old children (Ibanez et al., 2011).

Metformin exerts a direct inhibitory effect on the synthesis of ovarian steroid hormones and indirectly suppresses testosterone production by inhibiting LH secretion. Lundgren et al. conducted a study to assess the potential of metformin in enhancing the sensitivity of the hypothalamus to P4 in adolescent patients with HA during puberty. The findings of the study indicated that, while metformin was effective in improving biochemical hyperandrogenemia, it did not significantly enhance the sensitivity of the hypothalamus to P4 inhibition (Lundgren et al., 2018). Furthermore, Fontes et al. assessed the impact of metformin treatment on high testosterone levels in patients with PCOS. Experimental results showed that metformin effectively lowered total testosterone levels and free androgen index (Fontes et al., 2023).

Metformin exerts its efficacy by effectively reducing blood glucose levels, mitigating IR, and facilitating improvements in abnormal glucose metabolism markers. Through the inhibition of adipocyte fat breakdown and subsequent reduction in free fatty acid release, metformin contributes to improved lipid metabolism. Consequently, this mechanism may underlie the weight reduction observed in obese patients with PCOS. Rsearchers reported that metformin primarily ameliorates LH levels in lean patients with PCOS, whereas in obese patients with PCOS, its predominant effect lies in improving IR (Yang et al., 2018; Erensoy et al., 2019). Jensterle et al. discovered that overweight and obese patients with PCOS, after long-term metformin treatment, experienced significant reductions in basal metabolic rates, testosterone levels, and diabetes conversion rates (Jensterle et al., 2020). Therefore, both non-obese and obese patients with PCOS would benefit from metformin treatment.

In conclusion, metformin provides numerous advantageous effects on the health of adolescent patients with PCOS, including improvements in obesity, hirsutism, acne symptoms, insulin resistance, and other relevant markers. These enhancements lead to increased insulin sensitivity, decreased levels of insulin and testosterone, reduction of vascular inflammation, and optimization of lipid metabolism, ultimately aiding in the restoration of ovulatory function in affected individuals. Administering metformin as a treatment for adolescent PCOS, particularly during late childhood and early adolescence, has shown promising results in improving abdominal and hepatic fat metabolism. Additionally, metformin monotherapy may be a suitable alternative for adolescent girls who are reluctant to use oral contraceptives. However, studies indicate that metformin for adolescents may increase the incidence of mild to severe gastrointestinal side effects, such as nausea, vomiting, diarrhea, and flatulence. These side effects may affect quality of life and treatment adherence in adolescent patients (Meyers et al., 2021). In adolescent patients with PCOS, these side effects may have a noteworthy impact on efficacy.

### 3.2 Impact of metformin on pregnancy in patients with polycystic ovary syndrome

PCOS is a commonly occurring endocrine disorder that primarily affects females of reproductive age and is recognized as one of the leading causes of anovulation and female infertility. Those afflicted with PCOS frequently encounter difficulties in achieving natural conception. Despite successful conception achieved through medication, surgery, or assisted reproductive technology, patients may face various challenges such as early pregnancy miscarriage, gestational diabetes mellitus (GDM), gestational hypertension, and preeclampsia. Metformin improves IR, reduces HA and promotes weight loss, and plays a crucial role in regulating the menstrual cycle and facilitating ovulation. Nonetheless, the effectiveness, safety, and advantages of prolonged metformin use during pregnancy in patients with PCOS remain a topic of significant debate in the medical field.

#### 3.2.1 Impact of metformin on GDM in patients with polycystic ovary syndrome

GDM is one of the most common pregnancy complications in patients with PCOS, with significant implications for both the mother and fetus. Studies have found that females with PCOS have a threefold increased risk of developing GDM compared to those without PCOS, with a significantly higher risk for women with the hyperandrogenic PCOS phenotype (Hanna et al., 2023; Yan et al., 2022). IR is considered an underlying factor in PCOS, playing a crucial role in its pathogenesis. IR predisposes patients with PCOS to abnormal blood glucose levels, type 2 diabetes, and cardiovascular disease. During pregnancy, pre-existing IR and impaired glucose tolerance in patients with PCOS worsen, leading to an increased risk of GDM (Amisi, 2022; Ding et al., 2021). Pre-pregnancy hyperinsulinemia and glucose abnormalities may also affect endometrial receptivity, reducing fertility and increasing the risk of pregnancy failure in patients with PCOS. Metformin is the first-line medication for PCOS patients with IR. It activates AMPK to induce a shift from anabolic to catabolic metabolism, increases insulin sensitivity, and promotes the secretion of growth/differentiation factors in the intestines and kidneys, thereby reducing dietary intake and lowering blood sugar and weight during pregnancy in patients with PCOS (Dunne et al., 2023; Tocci et al., 2023). Doi et al. found that taking metformin in early pregnancy could reduce the risk of GDM (Doi et al., 2020). Similarly, other researchers have reported that, compared to insulin, metformin treatment better controls postprandial blood sugar during pregnancy reduces the risk of hypoglycemia, and limits maternal weight gain, thereby preventing the onset of GDM in pregnancies with PCOS (Picon-Cesar et al., 2021). In addition, studies have shown that metformin appeared to be effective and safe in patients with GDM, especially in overweight women. Compared to insulin therapy, metformin treatment for GDM shows potential advantages in terms of weight gain and neonatal outcomes (Simmons, 2010; Lautatzis et al., 2013). However, metformin treatment throughout pregnancy may result in reduced weight gain and lower insulin levels in patients with GDM (Newman and Dunne, 2022). In addition, due to the ability of metformin to cross the placenta, continued use of metformin for GDM during pregnancy still raises concerns about its safety and potential adverse effects on the fetus.

#### 3.2.2 Impact of metformin on patients with polycystic ovary syndrome experiencing pregnancy-induced hypertension

Hypertensive disorder of pregnancy is one of the causes of perinatal maternal death and seriously threatens the health of mothers and neonates. The incidence of hypertension in patients with PCOS can be as high as 13.1%, which is 1.75 times higher than that in non-PCOS patients (Garovic et al., 2022; Glintborg et al., 2018). A meta-analysis involving 6,078 subjects revealed that both systolic and diastolic blood pressure were elevated in patients with PCOS compared to non-PCOS individuals, with a more pronounced increase observed in those with a body mass index (BMI) of 25–30 kg/m^2^ (Zhuang et al., 2022). HA and IR can exacerbate endothelial damage and disrupt endothelium-dependent vasodilation mechanisms, leading to vascular smooth muscle hypertrophy and lipid metabolism disorders (Krentowska and Kowalska, 2022; Wang et al., 2019). Therefore, IR, HA and obesity are the main factors contributing to the increased risk of pregnancy-induced hypertension in patients with PCOS. Elevated levels of free testosterone may induce heightened sympathetic nervous system activity and vascular reactivity, promoting the onset and progression of pregnancy-induced hypertension. Additionally, some patients with PCOS undergo ovulation induction treatments such as assisted reproductive technology (ART), leading to an increased incidence of twin or multiple pregnancies, which may further elevate the risk of pregnancy-induced hypertension (Neven et al., 2022; Xue et al., 2022; Dai et al., 2023; Ban et al., 2024).

Research has shown that metformin improves HA, menstrual status, and ovarian blood flow, restores ovulation, and increases the pregnancy rate in infertile patients with PCOS. Metformin also enhances endothelial function, improves lipid metabolism, reduces the intima-media thickness of the carotid artery, and lowers the risk of cardiovascular diseases such as hypertension (Poniedziałek-Czajkowska et al., 2021). Kalafat’s meta-analysis found that taking metformin during pregnancy significantly reduced the risk of hypertensive disease and diabetes. The rates of prevention for preeclampsia, pregnancy-induced hypertension, and GDM were 92.7%, 92.8%, and 99.2%, respectively, compared with any other treatment or placebo (Kalafat et al., 2018).

#### 3.2.3 Impact of metformin on pregnant patients with polycystic ovary syndrome and preeclampsia

Research has shown that metformin can inhibit the placental secretion of soluble fms-like tyrosine kinase 1 and promote arterial dilation, which suggests metformin may be a potential preventive and therapeutic agent for preeclampsia. In studies investigating the extension of gestational weeks in pregnant females with early-onset preeclampsia treated with metformin, metformin (1,000–3,000 mg/d) significantly prolonged the expected time for treatment, and shortened the length of stay in the neonatal intensive care unit. More importantly, this had no significant effect on maternal, fetal, or neonatal prognosis (McDougall et al., 2022). Furthermore, research has found that although metformin did not reduce the incidence of post-term births, it significantly reduced the incidence of prodromal preeclampsia and helped to limit maternal weight gain. This may be attributed to the role of metformin in reducing the production of sFlt-1 and soluble endoglin by endothelial cells, trophoblasts, and syncytiotrophoblasts, and modulating anti-angiogenic factors at the mitochondrial level (Cluver et al., 2021; Romero et al., 2017). In clinical practice, for pregnant females with PCOS at high risk of preeclampsia, daily low-dose aspirin (50–150 mg) can be initiated in early to mid-pregnancy, with the timing of medication determined based on individual factors. Prophylactic use can be maintained until 26–36 weeks of gestation or even until 34–36 weeks. Nevertheless, regarding the potential benefits of metformin in the treatment of preeclampsia, more research data need to be accumulated for further evaluation.

### 3.3 Impact of metformin on offspring birth outcomes in patients with polycystic ovary syndrome

Certain researchers posit that metformin undergoes absorption and clearance via a sequence of transport channels within the body. Initially, the drug enters cells through the plasma membrane monoamine transporter (PMAT) and organic cation transporter 3 (OCT3) located on the surface of small intestinal epithelial cells. Subsequently, metformin enters the bloodstream through OCT2 and eventually reaches the liver via OCT1, OCT2, and OCT3. As pregnancy advances and the placenta develops, the expression of OCT2 in the placenta increases, which facilitates the transport of metformin through these channels. Additionally, placental trophoblast cells express P-glycoprotein (P-GP), which enables further entry of metformin into the placenta. Therefore, the potential effects on offspring when metformin is used by pregnant females with PCOS should be thoroughly evaluated (Nguyen et al., 2018; Foretz et al., 2023).

#### 3.3.1 Effect of metformin on early pregnancy in patients with polycystic ovary syndrome

Patients with PCOS may be at a heightened risk of experiencing miscarriages during the early stages of pregnancy. There is ongoing debate regarding the specific independent risk factors associated with early miscarriage in PCOS. Current research suggests that HA/IR, obesity, and elevated LH levels are significant contributors to recurrent spontaneous abortion (RSA) in patients with PCOS (Liu et al., 2021). Insulin plays a crucial role in this process by directly impacting theca cells, increasing androgen secretion, stimulating the release of plasminogen activator inhibitor-1, and inhibiting plasmin production. These mechanisms can lead to placental thrombosis and ultimately result in miscarriage. Long-term high levels of LH inhibit the function of FSH, leading to luteinization of ovarian granulosa cells and premature follicle maturation, thereby increasing the risk of early miscarriage (Burchall et al., 2019). Metformin has been shown to potentially mitigate the risk of early pregnancy loss through modulation of reproductive hormone levels, specifically androgens and estrogens, resulting in the regulation of ovarian function. Al-Biate et al. conducted a prospective cohort study to assess the efficacy of metformin treatment in reducing early pregnancy miscarriage among pregnant females with PCOS. The study revealed a significantly lower early pregnancy loss rate of 8.9% (5/56) in the metformin group compared with 36% (18/50) in the control group (Al-Biate, 2015). Furthermore, Løvvik et al. found that metformin may improve preterm and late miscarriages by inhibiting mammalian target of the rapamycin complex 1 (mTORC1) protein signaling, but its preventive effect on gestational diabetes was limited (Lovvik et al., 2019). Furthermore, a meta-analysis encompassing 13 studies comprising 1,606 females diagnosed with PCOS revealed that sustained administration of metformin leads to a notable decrease in miscarriage and preterm birth rates, alongside an increase in full-term pregnancies. Metformin treatment throughout pregnancy in women with PCOS does not cause significant adverse effects such as fetal growth retardation or intrauterine death (Lautatzis et al., 2013). Consequently, the continuous use of metformin during pregnancy among females with PCOS may serve as a beneficial approach to enhancing the likelihood of full-term pregnancy while mitigating the risks associated with early miscarriage and preterm delivery.

#### 3.3.2 Effect of metformin on offspring malformations in patients with polycystic ovary syndrome

The period of early pregnancy is critical for organogenesis and the potential teratogenic effects of drugs. The potential impact of metformin exposure on the incidence of fetal congenital malformations warrants careful consideration. It has been observed that metformin accumulates significantly in the fetus and placenta through the OCT. Hence, it can be concluded that fetal metformin concentrations closely mirror those found in maternal circulation. During the initial trimester of pregnancy, fetal mitochondria exhibit a degree of immaturity and reduced expression of OCTs, suggesting that the administration of metformin in the first trimester is associated with a relatively low-risk profile. Cassina et al. conducted a meta-analysis examining the effects of metformin treatment in early pregnancy on populations. The study included 517 patients with PCOS who ceased metformin treatment upon confirmation of pregnancy, with a recorded birth defect rate of 0.6%. In comparison, 634 patients with PCOS who continued metformin treatment during early pregnancy exhibited a birth defect rate of 0.5% (Cassina et al., 2014). At present, there is a lack of evidence to suggest a heightened risk of birth defects in the offspring of females with PCOS who utilized metformin during the initial stages of pregnancy. Likewise, previous studies have indicated that the administration of metformin in early pregnancy does not elevate the likelihood of non-hereditary congenital abnormalities. There were no significant teratogenic effects associated with treatment with metformin during early pregnancy in patients with PCOS (Newman and Dunne, 2022). Although the safety of metformin use in early pregnancy has been tentatively affirmed, additional data are required to definitively confirm its safety profile.

#### 3.3.3 Effect of metformin on birth weight in offspring of patients with polycystic ovary syndrome

The progression of the placenta and fetus necessitates a heightened oxygen demand and the presence of fully developed mitochondria in the later stages of pregnancy to facilitate the transportation of essential nutrients for fetal development (Joshi et al., 2022). Nonetheless, the administration of metformin hampers the functionality of the electron transport chain within mitochondrial respiratory complexes, resulting in diminished adenosine triphosphate (ATP) synthesis. Additionally, metformin has the potential to impede growth, impact pancreatic β-cells, and hinder glycolysis and the tricarboxylic acid cycle, potentially influencing fetal growth and cellular differentiation. Furthermore, it is important to consider that both relative nutrient restriction *in utero* and drug exposure may play a role in the development of obesity. Various clinical trials conducted by researchers have yielded diverse findings on this issue (Scherneck et al., 2018). Syngelaki et al. conducted a comparative trial examining the effects of metformin versus placebo on maternal weight gain in obese, non-diabetic pregnant females. The study found that maternal weight gain was significantly lower in the metformin group compared to the placebo group, with no significant difference observed in newborn birth weight. Specifically, in non-diabetic females with a BMI exceeding 35 kg/m^2^, prenatal administration of metformin resulted in reduced maternal weight gain without impacting newborn birth weight (Syngelaki et al., 2016). Previous studies have indicated that metformin administration during pregnancy has shown limited impact on fetal growth and development in patients with PCOS but may result in increased head circumference in the offspring of overweight mothers (Hjorth-Ha et al., 2018). Furthermore, these findings have been corroborated by additional research (Landi et al., 2019; Hanem et al., 2019).

#### 3.3.4 Effect of metformin on neonatal hypoglycemia in patients with polycystic ovary syndrome

Research has indicated that maternal administration of metformin in the later stages of pregnancy can result in fetal exposure to the drug. Neonatal blood concentrations of metformin have been found to equal or surpass half of maternal levels, raising concerns about the potential impact of elevated metformin levels on neonatal glucose metabolism and the development of hypoglycemia in newborns. Maternal hyperglycemia is identified as a significant risk factor for neonatal hypoglycemia. In a meta-analysis examining the efficacy of metformin treatment for GDM, it was found that the incidence of severe neonatal hypoglycemia was significantly reduced in the metformin-treated GDM group, potentially due to improved maternal glycemic management (Flory and Lipska, 2019; Abolhassani et al., 2023). In normoglycemic pregnant females with PCOS, exposure to metformin was not associated with an increased risk of neonatal hypoglycemia (Feig et al., 2020; Brand et al., 2022). These findings demonstrate that the incidence of neonatal hypoglycemia is predominantly influenced by maternal glycemic control, rather than by metformin exposure (Nilsen et al., 2024; Sheng et al., 2023).

**FIGURE 3:**
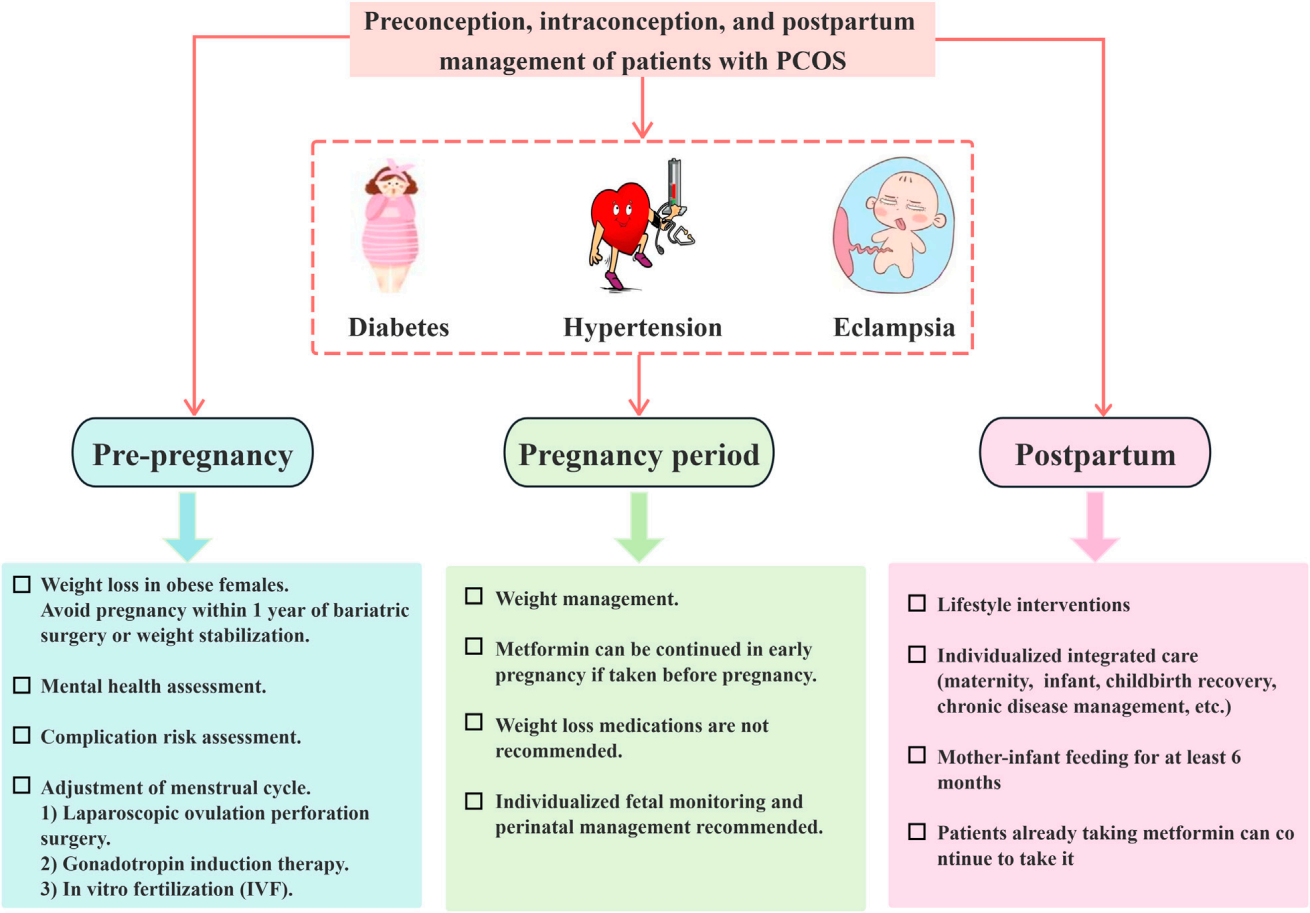
Management of patients with polycystic ovary syndrome throughout the preconception, gestational, and postpartum periods.

In summary, comprehensive care encompassing preconception, prenatal, and postnatal stages is essential for individuals with PCOS. Weight management and lifestyle modifications are fundamental components of pregnancy care for this population, as depicted in Figure 3, management of patients with PCOS before, during, and after pregnancy. Moreover, it is imperative to prioritize the assessment of mental health and its related complications. A meta-analysis of metformin use during pregnancy for the treatment of diabetes mellitus combined with PCOS showed that early exposure to metformin does not increase the risk of fetal birth defects (Paschou et al., 2024).

The administration of metformin in early pregnancy has been shown to enhance the likelihood of full-term pregnancies while decreasing the incidence of early miscarriage and preterm birth. Additionally, it has been observed that maternal weight loss with metformin does not have a significant impact on newborn birth weight (Feig et al., 2023). However, the use of metformin by overweight mothers may result in larger offspring head circumference. While there may not be notable disparities in offspring birth height and weight, there is a possibility of “catch-up growth” occurring at a later stage, the long-term implications of which remain uncertain until further long-term follow-up studies are conducted.

### 3.4 Long-term effects of metformin in patients with polycystic ovary syndrome

In addition to reproductive symptoms, females diagnosed with PCOS typically present with a variety of systemic symptoms. These individuals may experience various complications, such as cardiovascular disease (including type 2 diabetes, hypertension, and hyperlipidemia), metabolic dysfunction-associated steatotic liver disease (MASLD), metabolic syndrome (MS), and endometrial cancer (EC) (Cooney and Dokras, 2018; Rena and Lang, 2018). Therefore, it is of great significance to pay close attention to the long-term health of patients with PCOS.

#### 3.4.1 Protective effects of metformin on cardiovascular disease risk in patients with polycystic ovary syndrome

Epidemiological research suggests that individuals with PCOS exhibit a greater prevalence of hypertension compared to those without PCOS, particularly among females of reproductive age, and their cardiovascular risk is notably elevated irrespective of their BMI. Metformin, initially utilized for the treatment of diabetes, has been discovered to have great cardiovascular benefits as well (Long et al., 2022; Cameron et al., 2016).

**FIGURE 4:**
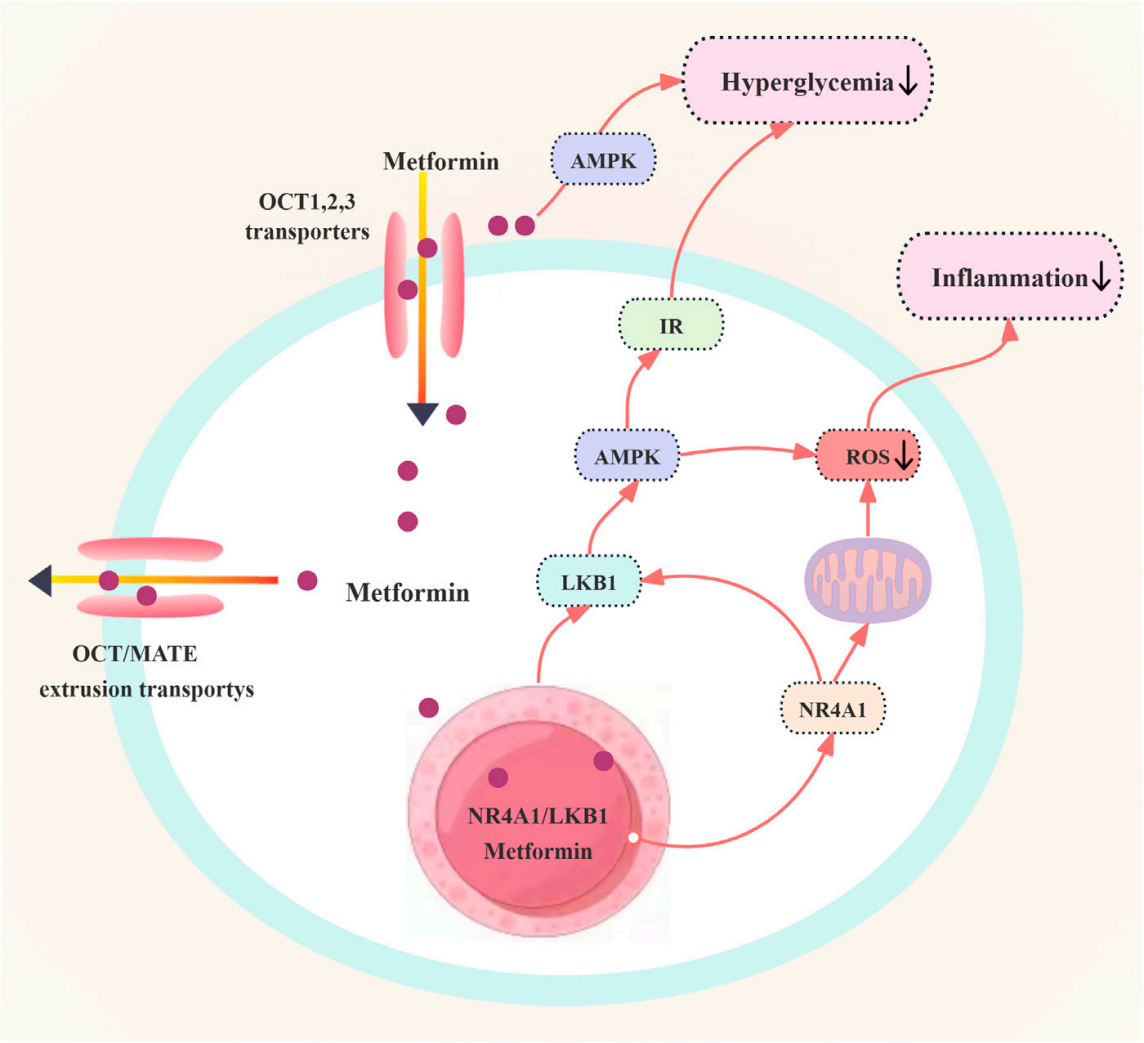
Regulation of blood glucose by metformin in patients with polycystic ovary syndrome. Abbreviations: OCT: organic cation transporter; IR: insulin resistance; AMPK: adenosine monophosphate-activated protein kinase; LKB1: liver kinase B1; ROS: reactive oxygen species; NR4A1: Nuclear receptor 4A1.

Metformin, originally prescribed for diabetic individuals, has been recognized for its significant cardiovascular protective properties. Research indicates that metformin can mitigate IR and enhance endothelium-dependent vasodilation (EDV). Investigations have revealed that the orphan nuclear receptor NR4A1 is pivotal in mediating metformin’s protective effects against endothelial dysfunction induced by high glucose levels. NR4A1 is capable of directly interacting with metformin, influencing the localization of Liver Kinase B1 (LKB1), activating AMPK, and decreasing the generation of inflammatory signals and intracellular ROS. Additionally, metformin can inhibit high glucose-induced NF-κB activation, which is also associated with increased AMPK phosphorylation (Zilov et al., 2019; Krug et al., 2019). The regulation of blood glucose by metformin in patients with PCOS is shown in Figure 4.

Hence, it is the prevailing recommendation among endocrinologists that non-pregnant individuals with PCOS exhibiting abnormal glucose regulation, irrespective of their body weight status, initiate metformin therapy promptly upon diagnosis, with a prescribed duration of treatment lasting at least 3–6 months. In the case of individuals intending to conceive, the use of metformin may be continued until pregnancy is confirmed, whereas for those not actively pursuing pregnancy, metformin may be administered until normalization of glucose regulation is achieved.

#### 3.4.2 Metformin improves metabolic dysfunction-associated steatotic liver disease in patients with polycystic ovary syndrome

MASLD is a prevalent liver disorder, with a reported prevalence of 14.5%–77% in patients with PCOS. Research indicates that the co-existence of PCOS and MASLD increases the likelihood of developing type 2 diabetes, cardiovascular disease, and chronic kidney disease, which are more serious and difficult to manage than MASLD alone (Shahbaz et al., 2022). Metformin functions by activating AMPK, which inhibits hepatic lipid synthesis, enhances hepatic fatty acid oxidation, facilitates the phosphorylation of target substrates, and supports mitochondrial function and adipose tissue integrity. These pathways collectively contribute to the mitigation of the initiation and progression of MASLD (Chen et al., 2017; Jang et al., 2023). Metformin treatment not only significantly improves metabolic and endocrine markers in patients with PCOS but also achieves a reduction of more than 10% in parameters such as weight, serum alanine aminotransferase (ALT), and gamma-glutamyl transferase (GGT) levels (de Oliveira et al., 2019; Ruan et al., 2023; Guan et al., 2020; Riemann et al., 2022). In obese individuals with PCOS, a notable decrease in weight, ALT, and GGT levels was observed following an 8-month course of metformin treatment. Additionally, in young overweight patients with IR and PCOS, a 12-month regimen of metformin therapy demonstrated a significant reduction in the prevalence of MS and amelioration of liver complications, enhancing insulin sensitivity (Hu et al., 2021).

Therefore, metformin has demonstrated efficacy in the management of PCOS in conjunction with MASLD, particularly in overweight and obese females with PCOS and IR. Overall, metformin is anticipated to mitigate liver fat accumulation and inflammation, potentially delaying the onset and progression of MASLD in individuals with PCOS. In cases where lifestyle modifications are insufficient to control weight and improve fatty liver, non-pregnant individuals with PCOS may benefit from early initiation of metformin therapy. A minimum treatment duration of 3–6 months is recommended to achieve weight loss goals.

#### 3.4.3 Metformin improves MS in patients with polycystic ovary syndrome

MS is a cluster of conditions, including central obesity, abnormal lipid metabolism, impaired glucose tolerance, and hypertension (Silveira et al., 2022). Individuals with PCOS are at an elevated risk of developing MS, particularly among adolescent patients. HA may serve as a common underlying mechanism for the co-occurrence of PCOS and MS (Da et al., 2020). Given the strong association between IR and PCOS, it is imperative to assess patients with PCOS for their susceptibility to MS (Zeng et al., 2020; Dobbie et al., 2023). Metformin exerts a crucial influence by inhibiting gluconeogenesis, glycogen synthesis, protein synthesis, and proliferation, as well as reducing fatty acid and cholesterol synthesis. A study conducted on patients with PCOS demonstrated significant improvements in BMI, low-density lipoprotein (LDL) cholesterol, and total cholesterol levels following treatment with metformin. Furthermore, Asemi et al. revealed that adherence to the Dietary Approaches to Stop Hypertension (DASH) diet for 8 weeks led to a significant decrease in serum insulin, triacylglycerol, and very low-density lipoprotein cholesterol (VLDL-C) levels, while increasing total antioxidant capacity (TAC) and glutathione (GSH) levels (Asemi et al., 2014). In addition, a study involving 140 hyperinsulinemic overweight females with PCOS found that metformin significantly decreased the prevalence of MS and enhanced liver function parameters. Moreover, menstrual regularity was restored to normal levels after a 12-months of metformin treatment (Gangale et al., 2011). Therefore, it can be concluded that metformin is an effective treatment for PCOS in conjunction with MS.

#### 3.4.4 Effects of metformin on EC in patients with polycystic ovary syndrome

**FIGURE 5:**
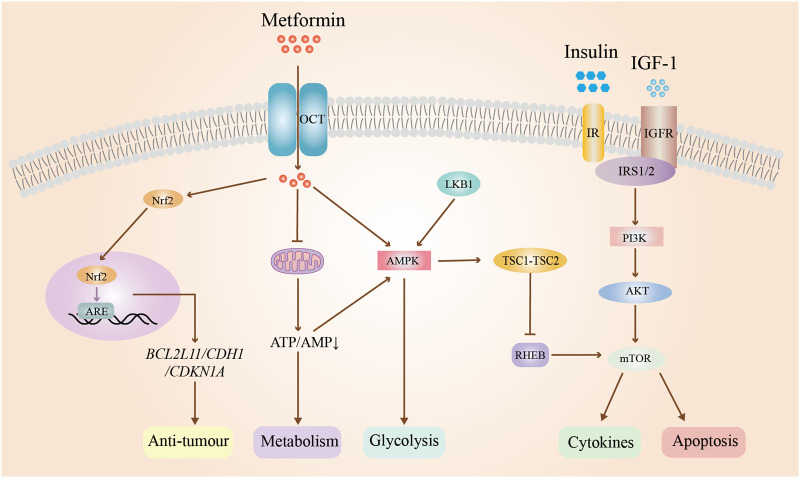
Mechanism of action of metformin on endometrial cancer. Abbreviations: OCT: organic cation transporter; IR: insulin resistance; IGF-1:insulin-like growth factor-1; IGFR: insulin-like growth factor receptor; Nrf2: nuclear factor erythroid 2-related factor 2; PI3K: phosphatidylinositol 3; AKT: protein kinase B; AMPK: adenosine monophosphate-activated protein kinase; ARE: androgen response element; ATP: adenosine triphosphate; AMP: adenosine monophosphate; RHEB: ras homolog enriched in brain; mTOR: mammalian target of rapamycin. TSC1-TSC2: tuberous sclerosis complex. BLC2L11: protein coding gene; CDH1: Cadherin 1; CDKN1A: cyclin-dependent kinase inhibitor 1A.

EC ranks as the second most prevalent gynaecological tumor, presenting with clinical symptoms including irregular vaginal bleeding, menorrhagia, or infertility (Xue et al., 2021). While early-stage EC can be effectively treated through surgical intervention or a combination of radiotherapy, a subset of patients seeks non-surgical treatment options, particularly those desiring to preserve fertility. Progesterone is typically the initial pharmacological choice for conservative management of early EC. Nevertheless, the administration of high doses of progestins may lead to adverse effects such as thrombosis, hyperglycemia, and edema. Additionally, approximately 30% of patients with PCOS and EC experience ineffective treatment due to resistance to progestins. Recent research has demonstrated that metformin may extend disease-free and overall survival in patients with EC. The potential mechanisms underlying this effect include the drug’s water solubility, which facilitates its passage through cell membranes, as well as its uptake via OCTs and excretion with the assistance of multidrug and toxin extrusion (MATE) proteins. Additionally, metformin has been shown to upregulate the expression of GLUT4 in endometrial cells of patients with PCOS, thereby inhibiting the progression of EC (Li et al., 2023). Simultaneously, metformin activates the LKB1/AMPK signaling pathway, leading to the inhibition of aerobic glycolysis in cells, as well as the insulin-like growth factor-1(IGF-1) signaling pathway and mTOR pathway in EC cells. This results in the suppression of cell proliferation, as well as the induction of cell cycle arrest and apoptosis (Zhou et al., 2024). Liu et al. demonstrated that the co-administration of Diane-35 and metformin resulted in decreased androgen receptor expression, increased GLUT4 expression, and modulation of cellular autophagy initiation by inhibiting IGF-1, ultimately leading to the suppression of EC development mediated by the mTORC pathway (Liu Y. et al., 2022). Furthermore, metformin regulates fatty acid (FA) synthesis through the AMPK/SREBP-1 pathway, thereby reducing the likelihood of malignant transformation in cancer cells (Zhou et al., 2024; Thüner et al., 2021). Metformin modulates the mTOR pathway and impacts the expression of particular proteins to elicit anti-tumor effects. A study revealed that metformin treatment induced temporal alterations in the expression of specific genes (*BCL2L11*, *CDH1*, and *CDKN1A*) and proteins in EC cell lines, demonstrating its anti-tumor and anti-proliferative properties in individuals with EC and PCOS (Lange et al., 2021). In summary, metformin may affect EC through the modulation of glycolysis, lipogenesis, and protein synthesis, as well as inhibition of tumor cell growth and proliferation, and promotion of apoptosis. The mechanism of action of metformin on EC is shown in Figure 5.

In summary, metformin demonstrates limited efficacy in the prevention of EC associated with PCOS. The management of patients with PCOS requires a systematic and meticulous approach. In clinical practice, it is essential to actively monitor patient’s hormone, insulin, and blood sugar levels, as well as closely observe the endometrial condition through regular examinations and curettage to detect abnormal hyperplasia at an early stage (Wang et al., 2022). Additionally, implementing appropriate dietary adjustments and addressing endocrine disorders are crucial components in the prevention of EC.

## 4 Discussion

PCOS is the predominant endocrine disorder affecting reproductive-aged women worldwide. Clinically, it presents with significant heterogeneity and has enduring implications for women’s health. Current research suggests that the pathogenesis of PCOS is intricately linked to factors such as genetics, environment, IR, and dysfunction of the HPOA. As understanding of PCOS evolves, IR is increasingly recognized as a key factor in the pathogenesis of the disorder, resulting in the widespread utilization of metformin, a medication that enhances insulin sensitivity, for the management of PCOS. More significantly, metformin has a meaningful preventative effect against cardiovascular disease and potential long-term complications of EC. This review systematically examines metformin’s therapeutic mechanisms in PCOS management, while assessing its clinical impacts across critical developmental phases—from pubertal maturation through gestation to long-term health maintenance. The findings provide valuable insights to optimize metformin’s therapeutic application in PCOS treatment, enabling safer pharmacologic management and improved patient prognoses.

Studies have demonstrated that metformin administration can enhance the autophagy, proliferation, and promote differentiation in granulosa cells while suppressing apoptotic pathways. Furthermore, it improves vascularization processes in PCOS, collectively supporting follicular maturation and ovulation induction. Additionally, metformin can improve endometrial receptivity by increasing the expression of GLUT4 in the endometrium, regulating the expression of hormone receptors related to endometrial decidualization, and modulating autophagy and nucleic acid/protein transcription processes, which in turn improves the reproductive outcomes of PCOS. Due to the ambiguous pathogenesis and the intricate interaction of multiple factors, the range of clinical treatment options for PCOS is notably restricted. Consequently, future studies must employ advanced methodologies, such as transcriptomics and proteomics technologies with high-throughput capabilities, to conduct more precise investigations into the underlying causes of suboptimal reproductive outcomes in patients with PCOS. This approach has the potential to facilitate the development of tailored treatment strategies aimed at addressing the complexities involved in enhancing reproductive outcomes for individuals diagnosed with PCOS.

The enduring impact of PCOS on women’s health necessitates a nuanced understanding of the varying efficacy of metformin treatment across different stages of the condition. As a primary therapeutic option for adolescent PCOS, metformin, whether used either as a standalone therapy or in conjunction with oral contraceptives, can effectively reduce androgen levels, alleviate hyperandrogenism symptoms, restore regular menstrual cycles, ameliorate IR, and reduce the likelihood of long-term cardiovascular complications. More importantly, metformin monotherapy may be particularly beneficial for patients with PCOS in late childhood and early adolescence, as well as those who are averse to oral contraceptives. Notably, the adverse effects caused by metformin therapy for adolescent patients with PCOS, especially gastrointestinal side effects, need to be a concern, as they may affect their quality of life and treatment adherence. Additionally, metformin has been shown to be effective in reducing pregnancy-related complications in patients with PCOS, including gestational diabetes, hypertension, and preeclampsia. Nevertheless, the utilization of metformin during pregnancy is typically determined following a collaborative evaluation of the potential risks and benefits by obstetricians and reproductive endocrinologists in current clinical practice. Research has demonstrated that metformin is capable of crossing the placenta, and maternal use of metformin during pregnancy has not been shown to elevate the likelihood of perinatal complications, congenital malformations, neonatal death, preterm birth, or neonatal hypoglycemia. However, metformin can cross the placenta into the fetus, which has raised concerns about its safety and potential adverse effects. The effects of metformin on the offspring of continued metformin therapy during pregnancy still need to be further evaluated. Consequently, forthcoming studies should prioritize the early detection and thorough evaluation of PCOS subtypes and symptoms, while also investigating the enduring consequences of metformin therapy for PCOS and its potential repercussions on the health of offspring.

PCOS represents a persistent endocrine-reproductive disorder whose progressive nature correlates with escalating risks of systemic health complications. Consequently, continued management and frequent evaluation for potential complications are essential for individuals with PCOS, even after fertility challenges have been resolved. Extensive research has indicated that metformin exhibits notable effectiveness in addressing PCOS-related complications, including cardiovascular diseases, MASLD, MS, and EC, underscoring its significant potential to enhance long-term health outcomes in individuals with PCOS. Currently, it is recommended that patients with PCOS undergo regular screenings for cardiovascular diseases and related tumors, especially those with risk factors such as central obesity, overweight, diabetes, and prediabetes. However, the side effects of long-term use of metformin in the patients with PCOS should not be ignored, which include gastrointestinal reactions, neurological adverse reactions, hypoglycemia, metabolic problems, and so on. Therefore, the potential risks that may be associated with the long-term use of metformin still need to be further studied and evaluated. The advantages and disadvantages of long-term use of metformin in the treatment of PCOS should be carefully weighed, and a personalized treatment plan should be developed according to the patient’s specific situation. Meanwhile, for the long-term management of patients with PCOS, in addition to medication, comprehensive interventions such as lifestyle modification, dietary control, and psychological support should be emphasized.

In conclusion, metformin has demonstrated many therapeutic advantages in the treatment of patients with PCOS across multiple life stages, including puberty, pregnancy, and long-term health management. However, several challenges persist in its application for PCOS treatment. For instance, the efficacy of metformin may vary among different patients with PCOS. In patients with multiple comorbidities, the efficacy and safety of metformin are not yet known. For patients with PCOS, long-term use of metformin may cause adverse reactions, and its safety still needs to be further evaluated. In addition, the interaction of metformin with other drugs used to treat PCOS requires noteworthy attention. In clinical practice, individualized drug regimens need to be developed based on patient-specific conditions, and therapeutic drug monitoring and individualized genetic testing are effective tools to increase the efficacy and decrease the adverse effects of metformin for PCOS therapy. Continued research efforts are expected to lead to broader adoption of metformin in clinical practice.

## Glossary

PCOS: Polycystic ovary syndrome
HPOA: Hypothalamic-pituitary-ovarian Axis
OHSS: Ovarian hyperstimulation syndrome
IR: Insulin resistance
LH: Luteinizing hormone
HA: Hyperandrogenemia
PCOM: Polycystic ovarian morphology
FSH: Follicle-stimulating hormone
COC: Combined oral contraceptives
FGCs: Follicular granulosa cells
mTOR: mammalian target of rapamycin
GDF-9: Growth Differentiation Factor 9
BMP-15: Bone morphogenetic protein 15
TGF-β: Transforming growth factor-β
NADPH: Nicotinamide adenine dinucleotide phosphate
MMP: Matrix metalloproteinase
AMPK: Adenosine monophosphate-activated protein kinase
miRNAs: Micro RNAs
GLUT4: Glucose transporter protein 4
P-GP: P-glycoprotein
PDGF: Platelet-derived growth factor
CTGF-1: Connective tissue growth factor 1
PI3K/AKT: Phosphatidylinositol 3-kinase/protein kinase
LIF/STAT3: Leukemia inhibitory factor/signal transduction
ROS: Reactive Oxygen Species
PP: Precocious pubarche
P4: Progesterone
E2: Estradiol
HOXA10: Homology box A10
BP: Bee pollen
OCT: Organic cation transporter
IGF-1: Insulin-like growth factor-1
MATEs: Multidrug toxin extrusion proteins
PGR: Progesterone
AGEs: Advanced glycation end products
RAGE: Receptor for advanced glycation end product
NF-κB: Nuclear factor kappa B
LPS: Lipopolysaccharide
ITGB3: Integrin beta-3
GLM: Glucose-lipid metabolism
EDV: Endothelium-dependent vasodilation
LBW: Low birth weight
AUB: Abnormal uterine bleeding
GDM: Gestational diabetes mellitus
BMI: Body mass index
PMAT: Plasma membrane monoamine transporter
RSA: Recurrent spontaneous abortion
mTORC1: mammalian target of the rapamycin complex 1
ATP: Adenosine triphosphate
MASLD: Metabolic dysfunction-associated steatotic liver disease
EC: Endometrial cancer
MS: Metabolic syndrome
NR4A1: Nuclear receptor
LKB1: Liver Kinase B1
FA: Fatty acid
ALT: Aminotransferase
GGT: Gamma-glutamyl transferase
